# A platform to reproducibly evaluate human colon permeability and damage

**DOI:** 10.1038/s41598-023-36020-8

**Published:** 2023-06-01

**Authors:** Elizabeth E. Marr, Thomas J. Mulhern, Michaela Welch, Philip Keegan, Celia Caballero-Franco, Bryce G. Johnson, Marion Kasaian, Hesham Azizgolshani, Timothy Petrie, Joseph Charest, Elizabeth Wiellette

**Affiliations:** 1 Draper, 555 Technology Sq., Cambridge, MA 02139 USA; 2 Pfizer Inflammation and Immunology, 1 Portland St., Cambridge, MA 02139 USA

**Keywords:** Biotechnology, Drug discovery, Gastroenterology

## Abstract

The intestinal epithelium comprises diverse cell types and executes many specialized functions as the primary interface between luminal contents and internal organs. A key function provided by the epithelium is maintenance of a barrier that protects the individual from pathogens, irritating luminal contents, and the microbiota. Disruption of this barrier can lead to inflammatory disease within the intestinal mucosa, and, in more severe cases, to sepsis. Animal models to study intestinal permeability are costly and not entirely predictive of human biology. Here we present a model of human colon barrier function that integrates primary human colon stem cells into Draper’s PREDICT96 microfluidic organ-on-chip platform to yield a high-throughput system appropriate to predict damage and healing of the human colon epithelial barrier. We have demonstrated pharmacologically induced barrier damage measured by both a high throughput molecular permeability assay and transepithelial resistance. Using these assays, we developed an Inflammatory Bowel Disease-relevant model through cytokine induced damage that can support studies of disease mechanisms and putative therapeutics.

## Introduction

Inflammatory Bowel Disease (IBD), including both Ulcerative colitis (UC) and Crohn’s Disease (CD), is increasing around the world, with an incidence rate in the United States of 1.3% of adults in 2015^1^. The disease burdens the healthcare system with direct and indirect costs together estimated at $15–30 Billion per year. In addition, intestinal diseases are associated with higher incidence of other severe conditions, including cardiovascular disease, neuropsychological disorders, and metabolic syndrome^2–5^. Treatment strategies focus on reducing the inflammatory response and include antibodies that target inflammatory cytokines and cell recruitment as well as immunomodulators such as aminosalicylic acid and steroids^6–8^. Newer drugs include molecules that inhibit the JAK/STAT signaling pathways, which control cellular response to inflammatory cytokines, and S1PR modulators, that affect aberrant leukocyte migration^8–11^.

These treatments address the inflammatory aspects of IBD, however IBD results from both inflammation of the mucosal tissue and damage to the mucosal barrier, wherein these two primary damage points create a positive feedback loop^12,13^. Even as inflammation is suppressed or barrier damage is repaired, the cycle can re-initiate, leading to a relapsing and remitting disease. Contributing causes of IBD span a broad range of factors, including genetic predisposition, environmental toxicity, and microbiome dysbiosis. The complexity of both causation and progression of disease pathology leads to challenges in characterizing and treating the disease^14,15^. Thus, in IBD drug discovery to date, most treatment strategies target immune cells and mitigation of the inflammatory response, rather than maintaining the epithelial barrier. A potential reason that current treatments have significant non-response or loss-of-response could be their focus on suppressing inflammatory response but failure to heal the mucosal barrier. In order to develop therapeutic treatments for restoring the epithelial barrier, relevant models of barrier function are needed^16–19^.

Tissue culture-based models of inflammatory bowel diseases typically involve damage to isolated colonocytes or immune cells and in more complex models include multi-cell type cultures and microphysiologic systems (MPS)^20^. Current in vitro models of human colon mucosa rely extensively on the culture of epithelial stem cells as spheroids, or differentiation of induced pluripotent stem cells into colon organoids^21,22^. These three-dimensional cultures recapitulate diverse epithelial biology but lack easy access to the lumen side of the monolayer, precluding assessment of barrier function, application of drugs, or other sampling of luminal contents. Alternatively, these spheroid or organoid cells can be dissociated and used in a two-dimensional architecture that provides apical or basal access to a monolayer. Initially these studies were performed in Transwell platforms, but more recently researchers have begun to utilize microfluidic technologies^23,24^. To improve the longevity of the monolayer culture and enhance relevant cell physiology, MPS introduce environmental stimuli such as flow, stretch and physical patterning. Published colon MPS models using primary human cells have demonstrated relevant mucus production and, barrier function, as well as enhancedco-culture with T cells and relevant commensal and pathogenic microbes^25,26^. However, these examples are typically low throughput, high maintenance platforms, and identification of new therapeutic strategies will require extensive screening capability.

To meet the needs of high throughput drug discovery, we have developed a microfluidic platform, PREDICT96, that houses an array of 96 microscale devices on a single plate. Each device contains two chambers for cultured cells supplemented by recirculated media via an onboard pumping system. The top and bottom microfluidic chambers are separated by a porous membrane, allowing for paracrine communications between co-cultured cells and transport studies to be performed across single tissue layers^27–32^. We have previously demonstrated culture of primary human colon cells in PREDICT96-based tissue models, and in this study, we introduce a new capability to study the complexity of colon epithelial barrier function at a throughput sufficient to evaluate dose effects, time courses, and patient-specific sensitivity across many various treatments. Through this colon-on-chip model we demonstrate the capability to study epithelial barrier generation and damage by utilizing both integrated sensors to capture real time transepithelial electrical resistance (TEER) and a standardized molecular permeability assay. We demonstrate barrier function loss and recovery in response to damaging cytokines Tumor Necrosis Factor alpha (TNF-α) and Interferon gamma (IFN-γ), both recognized drivers of IBD in vivo, in a dose-dependent and reproducible manner^33^. The correlation of barrier damage with secretion of interleukin-8 (IL-8) provides confirmation of the damage response by the epithelial cells^34,35^. Individual tissue donor-derived cells demonstrate differential sensitivity to TNF-α, and we suggest that these assays are sensitive and precise enough to study variability of both toxicity and drug efficacy across a range of patient samples. We envision using the model of TNF-α or IFN-γ induced barrier damage as the basis to evaluate potential therapeutic compounds targeted at epithelial health to treat IBD.

## Results

### Establishment of differentiated colon micro-tissues in PREDICT96

**Figure 1:**
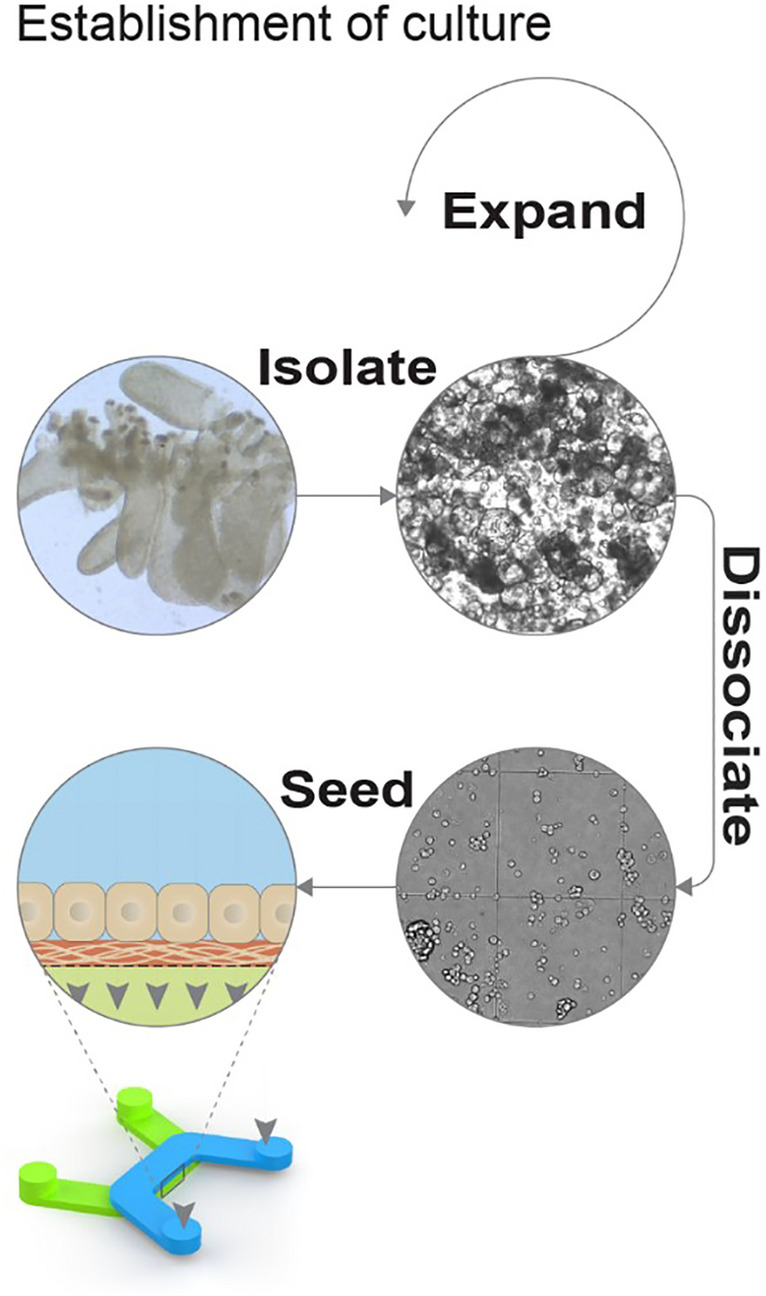
Establishment of human primary colon spheroid cultures and micro-tissues. Colon epithelial stem cells were isolated from the crypts of human intestine tissue using mechanical and enzymatic dissociation techniques and then plated in Matrigel to form primary 3D spheroid cultures. Spheroids were expanded and regularly passaged prior to seeding into devices. For device seeding, colon spheroids were dissociated into multi-cell aggregates, then delivered at high density into the top chamber of PREDICT96 plate bilayer devices utilizing pressure driven flow via a pipette seal press-fit interface.

**Figure 2:**
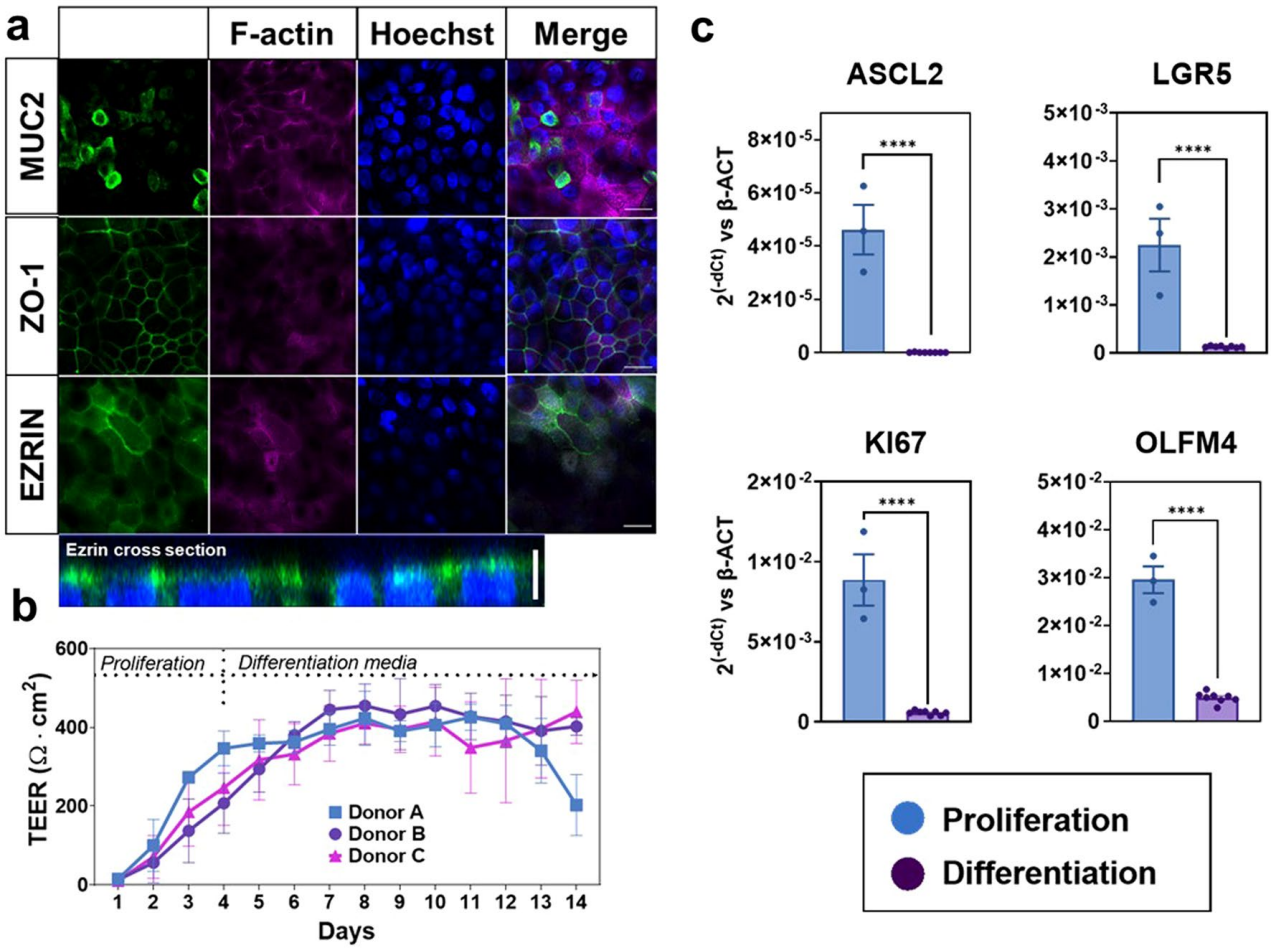
Mature and differentiated colon micro-tissues were consistently generated in microfluidic devices. (**a**) Representative images of colon micro-tissues that were fixed, stained and imaged within devices. Anti-MUC2 (mucus proteins), anti-ZO-1 (organized tight junction at cell borders), and anti-Ezrin (brush border at the apical surface of the tissue) were stained in green. All devices were co-stained with fluorescently labeled phalloidin to identify f-actin cytoskeletal protein (pink) and Hoechst 33342 to identify nuclei (blue). White scale bars represent 20 microns. Images are representative of data collected from 3 independent experiments. (**b**) Micro-tissue barrier function and health was monitored longitudinally by measuring TEER in micro-cultures derived from 3 different colon donors over 14 days. TEER data are representative of data collected from 3 independent experiments. (**c**) Differentiation was also monitored by changes in gene expression of key stem cell and proliferation markers (ASCL2, LGR5, KI67, OLFM4) cultured in DM compared to micro-tissues cultured in PM. Quantitative RT-PCR measurement of gene expression is reported as relative to expression of the housekeeping gene β-ACTIN for each sample. In all cases, error bars represent standard error of the mean. A two-tailed unpaired t-test was used to analyze tissues grown in PM versus DM with an α = 0.05 (* *p* < 0.05, ** *p* < 0.01, *** *p* < 0.005, **** *p* < 0.0001). N = 3 (IF imaging), N = 3–8 (PCR), N = 10 (TEER).

The previously introduced PREDICT96 platform supports high-throughput, multiplexed readouts of tissue models with integrated media recirculation and TEER measurements^27–32^. To provide a relevant model of colon permeability, damage, and repair, human primary colon epithelial cells were integrated into this device format. Following standard protocols for isolation and long-term culture of epithelial stem cells, human colon spheroid cultures were established from three deceased donor organs^24,36^ (Fig. 1). These cultures were used to seed intestinal stem cells into the PREDICT96 platform, where micro-tissues were established under static conditions in proliferation media and after 4 days transitioned to differentiation media. After 3 additional days in culture, colon tissue morphology was observed through robust and ubiquitous tight junction protein organization in zonula occludens-1 (ZO-1) staining, epithelial polarization in brush border protein (Ezrin) staining representative of the luminal surface in vivo, and mucin-2 (MUC2) protein localizing to mucus producing Goblet cells over all donors (Fig. 2A). The formation of epithelial barrier was further functionally demonstrated by tracking TEER, which reached a steady state at about 7 days and retained high TEER until at least day 10 and in many wells until day 14 (Fig. 2B). In isolated human colon tissue, TEER has been measured at about 300–400 Ω*cm^2^ in Ussing chambers^36^ and colon micro-tissues grown in PREDICT96 typically demonstrated TEER values of roughly 400 Ω*cm^2^. Once achieving high TEER at about 7 days post-seeding/3 days post-media type change, this resistance was maintained out to at least 14 days post-seeding for 2 out of three donors, although the third donor displayed decreased TEER from day 11 post-seeding (Fig. 2B). Differentiation was demonstrated by significant downregulation of key stem cell and proliferation marker genes ASCL2, LGR5, KI67, and OLFM4 (Fig. 2C; *p* < 0.0001). Moreover, as evidence of colon-like functional differentiation, gene expression components of complex mucus including MUC1, MUC2 and MUC4 were found to be significantly upregulated compared to micro-tissues similarly derived in PREDICT96 from ileum cultures (Supplementary Fig. S1, *p* = 0.0357, *p* = 0.0002, *p* = 0.0357). Genes specific to small intestine cell types such as APOA, REG3A, and SI were significantly downregulated in colon micro-tissues when compared to ileum-derived microtissues (Fig. S1, *p* = 0.0002, *p* = 0.0002, *p* = 0.0002, respectively) (see National Center for Biotechnology Information Gene Expression data). Taken together, these data characterizing protein expression, physiologically relevant barrier function, and tissue specific gene expression indicate reproducible generation of barrier forming human colon micro-tissues that contain relevant cell types in a format enabling multiplexed assay techniques.

### Colon barrier function damage measured by high-throughput electrical resistance measurements

**Figure 3:**
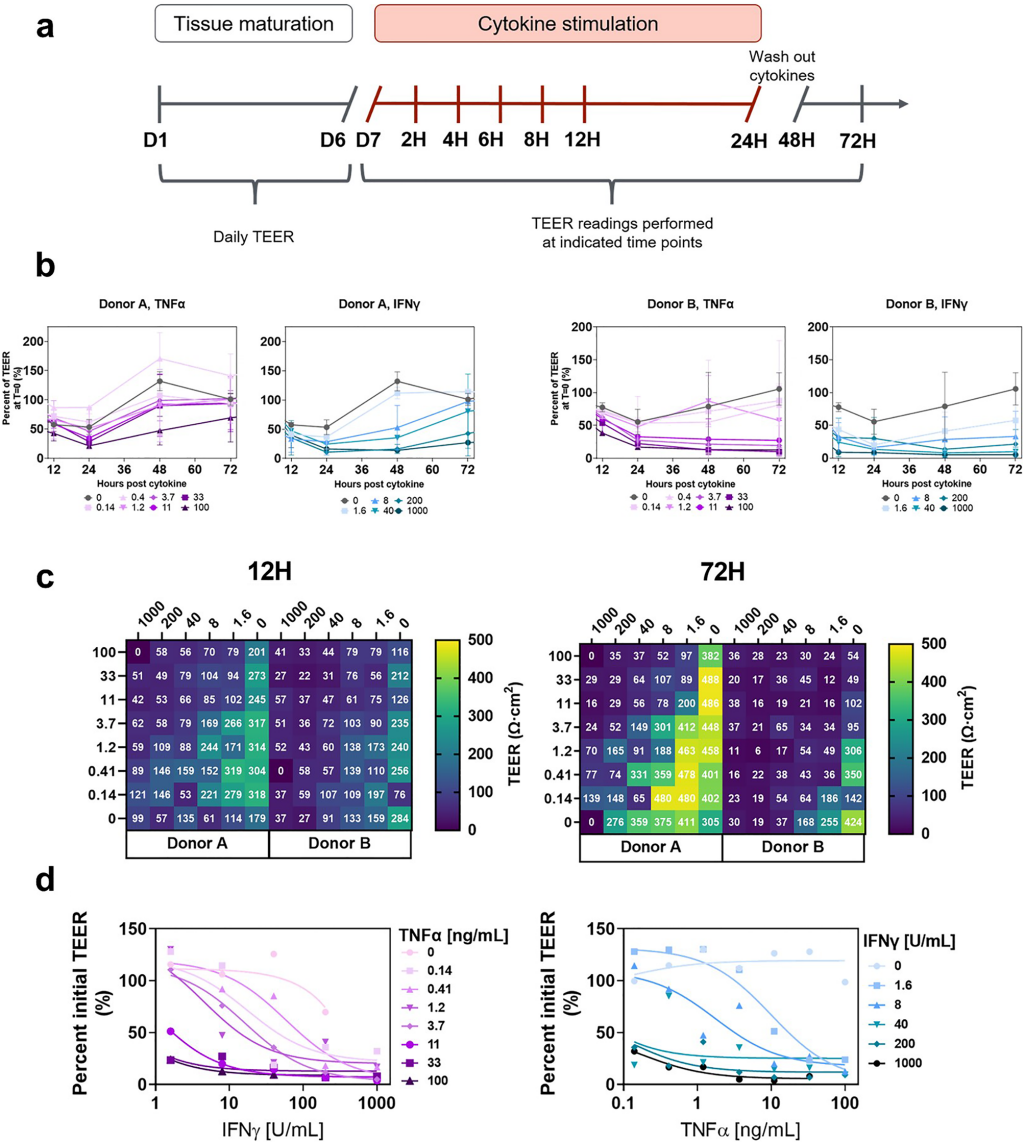
Precisely-controlled cytokine-induced colon barrier disruption model generated using high-throughput TEER screening. (**a**) Colon micro-tissues were grown in devices for 4 days in PM followed by 3 days in DM before being treated with cytokine doses for 24 h. Over the 24 h treatment window, TEER was regularly measured at 2, 4, 6, 8, 12, and 24 h post treatment. After 24 h, cytokine doses were washed out and replaced with cytokine-free differentiation media. Micro-tissue barrier function was tracked for 2 additional days via TEER measurements. (**b**) TEER values measured during the damage time course at 2 h intervals, and every 24 h during recovery. (**c**) Barrier disruption in response to combination dose curve of TNF-α (0.14–100 ng/mL) and IFN-γ (1.6–1000 U/mL). Heat map colors indicate relative TEER and white font reports exact TEER for each well. One representative experiment is reported during cytokine treatment (12 h) and after recovery (72 h) for two separate colon donors (Donor A, Donor B). (**d**) Dose curves were generated for TNF-α (left) and IFN-γ (right) for each of the sensitizing concentrations of IFN-γ and TNF-α. Data are displayed from Donor A after recovery (72 h after cytokine addition; 48 h after cytokine removal), where each data point was normalized to the pre-treatment TEER for comparison across wells.

IFN-γ and TNF-α are both implicated as secreted factors that damage the epithelial barrier function in IBD, and have been shown to damage epithelial cells in vitro^37–41^. Taking advantage of the large number of micro-tissues available in PREDICT96, we tested combined dose curves of IFN-γ and TNF-α across cultures derived from two separate donors. Cells from both donors established consistent TEER by day 5–6 post-seeding (Supplementary Fig. 2). At day 7 post-seeding, 48 unique cytokine dose pairs from a combined 6-dose curve of IFN-γ and an 8-dose curve of TNF-α were applied to healthy colon micro-tissues. After 24 h of exposure, the cytokines were washed out and TEER of micro-tissues was monitored for an additional 48 h (Fig. 3). The entire experiment was repeated twice and TEER measurements were collected throughout each experiment.

All micro-tissues, including the samples not exposed to cytokines, serving as controls—showed a decrease in TEER over the 24 h treatment window (Fig. 3). We hypothesize that the introduction and removal of cytokine-containing media paired with the temperature equilibration necessary for TEER measurements resulted in the temporary drop in TEER across all micro-tissues, including the untreated controls (Supplementary Fig. 2)^42^. While these handling effects are apparent during the 24-h treatment period, distinct responses to treatment conditions could be measured throughout the experiment. The combinatorial disrupting effect of IFN-γ and TNF-α can be observed qualitatively during the 24-h treatment period, with recovery of barrier resistance apparent by 72 h post-treatment (Fig. 3B; Supplementary Fig. 2). Colon micro-tissues treated with solvent control conditions fully recovered TEER after 48 h, while those challenged with high dose TNF-α combined with high dose IFN-γ did not recover barrier function after 48 h. Recovery of TEER in micro-cultures derived from Donor A was inversely dependent on the combination of IFN-γ and TNF-α. In contrast, recovery of TEER in micro-cultures derived from Donor B was more sensitive to TNF-α and IFN-γ, where lower doses of the cytokines proved sufficient to drive little to no recovery of TEER (Fig. 3c, Supplementary Fig. S3). This differential sensitivity to cytokines was consistent across repeat experiments. It is likely that the lack of recovery across nearly all Donor B- and some Donor A-derived micro-tissues is the result of extensive cell death given the recognized ability of these factors to drive cell death^43–45^. Viability was not measured directly at the endpoint, however recovery of TEER in many Donor A-derived micro-tissues indicates viable cells were present and able to re-establish an epithelial barrier. The data collected supports quantifiable characterization of barrier function by normalizing to each micro-tissue baseline with healthy TEER values prior to cytokine stimulation (reported as “percent initial TEER”). A non-linear, three parameter fit was performed on all data sets to correlate barrier function loss with exogenous cytokine concentration, and the half maximal concentration (EC_50_) of each cytokine needed to fully disrupt TEER was calculated (Fig. 3d, Table 1). This calculation was repeated across multiple IFN-γ or TNF-α doses and a shift in EC_50_ was observed with the inclusion of the additional cytokine (Fig. 3d, Table 1). Synergistic activity of IFN-γ and TNF-α was demonstrated in the shift in EC_50_ with increasing dose of the alternate factor. The high R^2^ values derived from the non-linear three parameter fit indicate sufficiently consistent data for these calculations (Table 1). Overall, these data demonstrate the ability of the PREDICT96 colon model to generate large data sets from a complex phenotypic assay using primary human cells, all within the framework of a single experiment.

**Table 1:** Synergistic effects of TNF-α and IFNg on barrier function evaluated by TEER.

Donor A, 72H: IFNγ EC50								
TNFα [ng/mL]	0	0.14	0.41	1.2	3.7	11	33	100
IFNγ EC50 [U/mL]	Unstable	18	59	4.5	16	0.96	0.21	0.33
R^2^	0.554	0.782	0.937	0.905	0.996	0.997	0.981	1

Donor A, 72 H: TNFα EC50								
IFNγ [U/mL]	0	1.6	8	40	200	1000		
TNFα EC50 [ng/mL]	0.14	10	1.7	0.03	0.12	0.18		
R^2^	0.430	0.941	0.845	0.676	0.801	0.902		

Non-linear three parameter fit was used to calculate EC_50_ values, shown with R^2^ values which correspond to Fig. 3C plots. “Unstable” was designated by Prism software and indicates that no inflection point was defined.

### Colon barrier function damage measured by high-throughput molecular permeability assay

**Figure 4:**
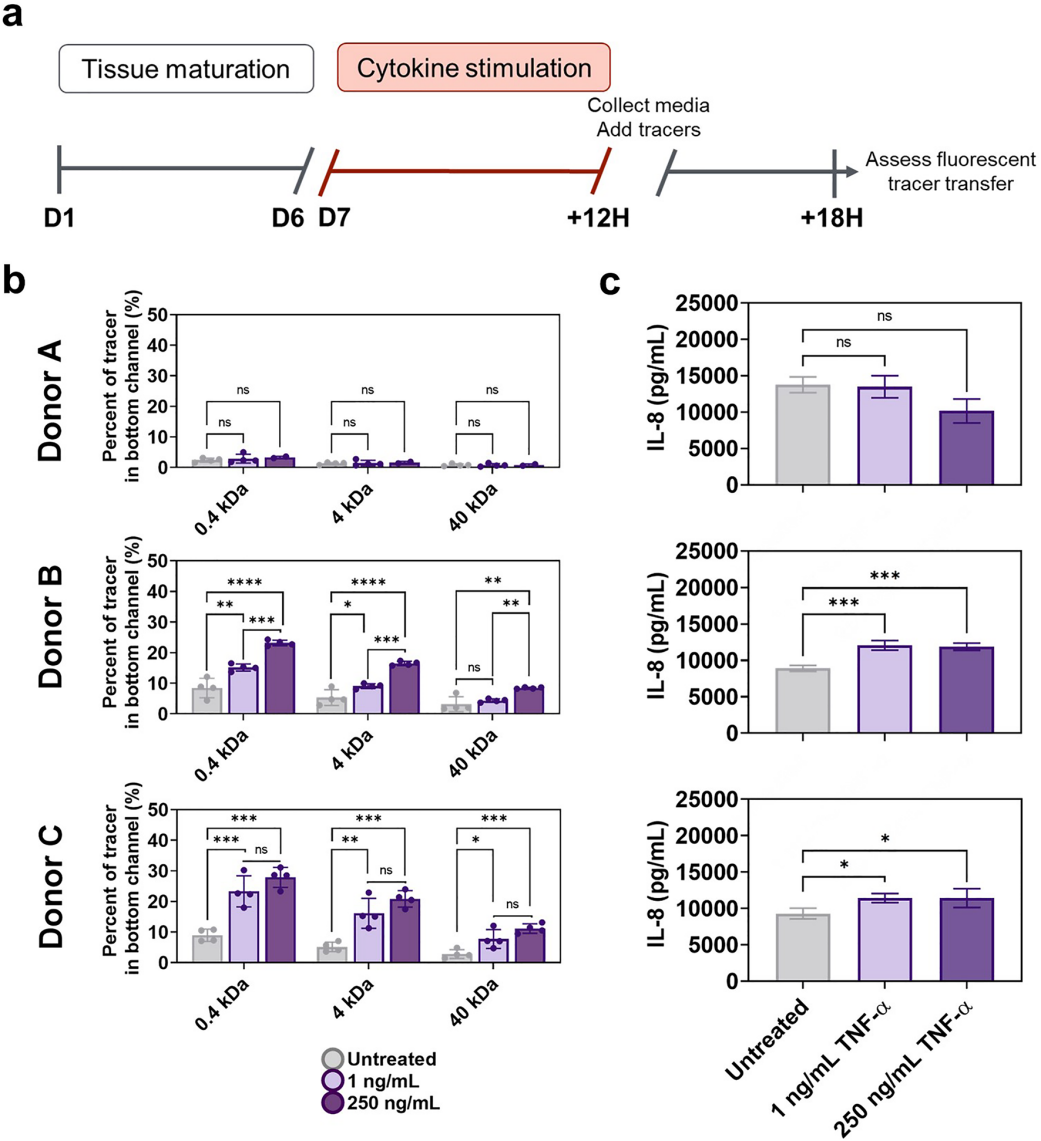
TNF-α dosing impacts colon micro-tissue permeability and IL-8 secretion (a) Colon tissues were generated in devices through 4 days culture in PM followed by 3 days in DM. Established colon micro-tissues (verified by high TEER) were exposed for 12 h to TNF-α doses and then replaced with the tracer dye molecules under recirculation. (**b**) Permeability was assayed as fluorescent tracer transfer from top to bottom channel after 6 h. Values are displayed as percent of tracer in bottom channel to quantify probe transfer across the epithelial barrier of micro-tissues derived from 3 colon donors. Results of 0.4 kDa lucifer yellow (LY), 4 kDa FITC-dextran and 40 kDa TRITC-dextran transfer are shown for high and low doses of TNF-α compared to untreated controls. All error bars represent standard error of the mean. A one-way ANOVA with α = 0.05 was utilized to compare dose responses within a group unique to each donor, cytokine dose curve, and tracer. Post hoc analyses were performed with Tukey’s multiple comparisons test after ANOVA. (ns not significant, * *p* < 0.05, ** *p* < 0.01, *** *p* < 0.005, **** *p* < 0.0001. N = 4). (**c**) Following damage to colon micro-tissues, media was collected from microfluidic chambers and IL-8 was measured using a commercial ELISA assay kit. All error bars represent standard error of the mean. A one-way ANOVA was performed with an α = 0.05 and Dunnett’s multiple comparisons test was used to compare cytokine stimulation conditions to the untreated control for each donor. (ns not significant, * *p* < 0.05, ** *p* < 0.01, *** *p* < 0.005, **** *p* < 0.0001. N = 3).

TEER measurements provide a rapid assessment of barrier function and evaluate cell–cell binding tightness as a function of permeability to electric current. However, the critical concern when studying barrier function in disease and toxicity conditions is whether the intestinal epithelium is permeable to molecules and bacteria^46–48^. To measure the physical permeability of the barrier, a high throughput functional assay was developed using the colon micro-tissues in PREDICT96. Healthy monolayers were treated at day 7 with cytokines for 12 h and upon wash out of test agents, tracer molecules were added in the top chamber (Fig. 4A). PREDICT96 pumps were applied to both top and bottom microfluidic chambers to support mixing throughout the sampling period, and samples were collected after 6 h following application of the tracer molecules (Fig. 4B). A mix of three different sized tracer molecules was used, Lucifer Yellow with a molecular weight of 0.4 kDa, FITC-dextran of 4 kDa, and TRITC-dextran at 40 kDa, potentially allowing discrimination of permeability to different sized molecules after 6 h of transfer. Consistently during these experiments, the smallest molecule, Lucifer Yellow, achieved maximal 30% transfer, while the 4 kDa dextran reached about 20% transfer and the 40 kDa dextran about 12% transfer, suggesting that size affects the rate or maximum achievable transfer.

The assay presented here provides a sensitive measurement of permeability of human epithelial micro-tissues, as demonstrated by reproducible variation in permeability among donors and tracer sizes. Cultures derived from three colon donors were assessed, and the established micro-tissues were treated with two doses each of TNF-α and IFN-γ, building on observed permeability changes in the TEER experiments. TNF-α exposure demonstrated the ability of the model and the assay to identify nuanced barrier disruption, considering both donor variability and tracer molecule size (Fig. 4B). In repeated experiments, Donor A did not demonstrate a permeable barrier after exposure to TNF-α, while Donors B and C were differently sensitive to this disruptor; these results parallel the TEER data which indicated Donor A is reproducibly more resilient to TNF-α exposure than Donor B (Supplementary Fig. S2). Micro-tissues derived from Donor B developed significant permeability to Lucifer Yellow in response to 1 ng/mL TNF-α and significantly higher permeability in response to 250 ng/mL (Fig. 4B). However, the larger molecules were not significantly permeable in cultures treated with 1 ng/mL TNF-α, and 40 kDa dextran was not transferred in cultures treated with 250 ng/mL TNF-α (Fig. 4B). Micro-tissues derived from Donor C were more sensitive to TNF-α, where 1 ng/mL was sufficient to maximize the permeability for Lucifer Yellow and 4 kDa dextran, and tissue treated with 250 ng/mL TNF-α was significantly permeable to 40 kDa dextran. For comparison, maximum transfer in acellular devices coated with Matrigel and run in parallel with cellular devices resulted in 39.1% ± 2.3% in bottom channel of lucifer yellow, 32.1% ± 2.4% in bottom channel of 4kD dextran and 22.6% ± 3.4% in bottom channel of 40kD dextran (n = 24). This assay thus provides a highly sensitive method to measure induction of permeability through primary human epithelial micro-tissues, evaluating both donor variability and size permeability in a single experiment.

**Figure 5:**
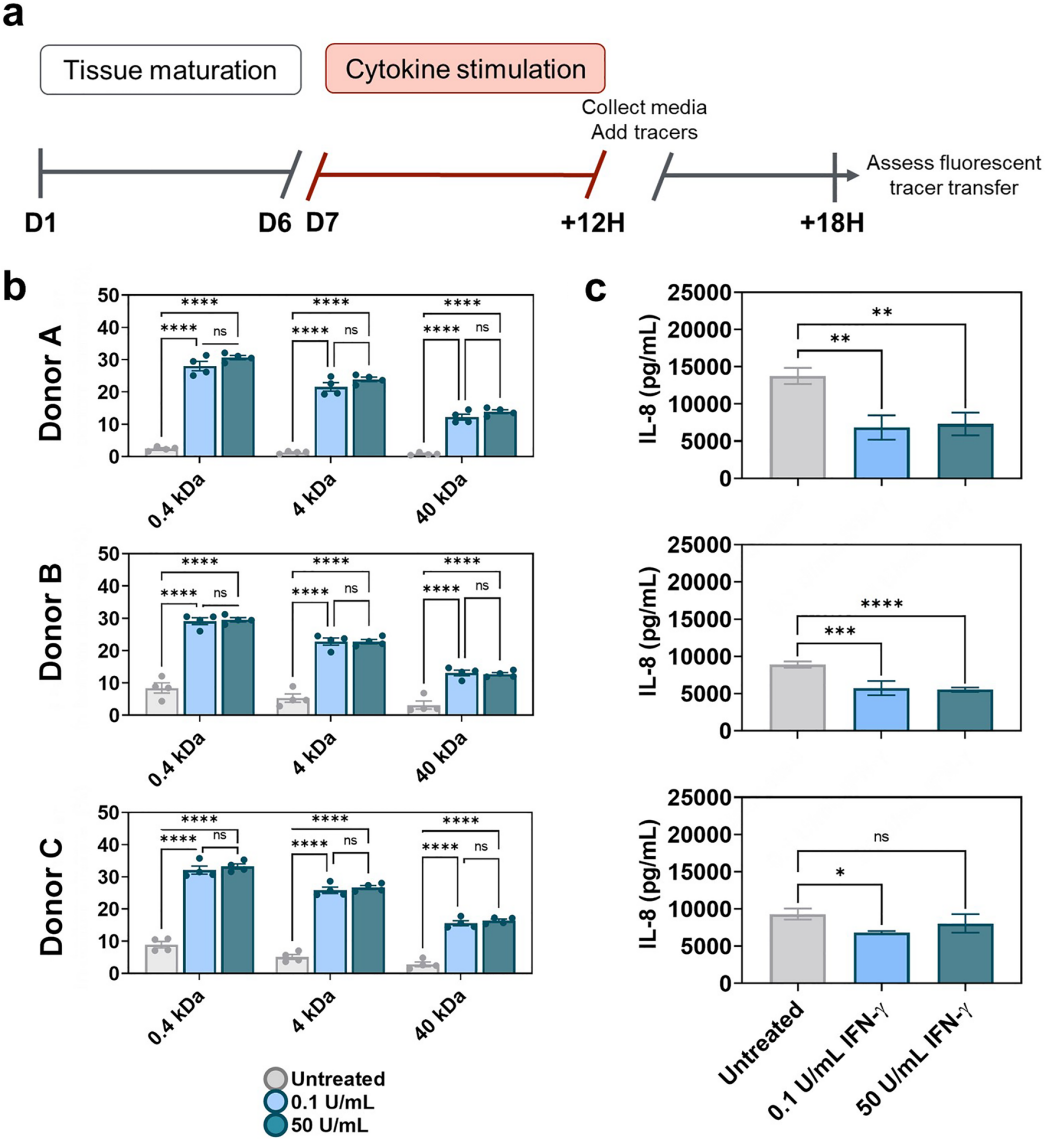
IFN-γ dosing impacts colon micro-tissue permeability and IL-8 secretion (**a**) Established colon micro-tissues (verified by high TEER) were exposed for 12 h to IFN-γ doses and then replaced with the tracer dye molecules under recirculation. (**b**) Permeability was assayed as fluorescent tracer transfer from top to bottom channel after 6 h. Values are displayed as percent of tracer in bottom channel to quantify probe transfer across the epithelial barrier of micro-tissues derived from 3 colon donors. Results of 0.4 kDa lucifer yellow (LY), 4 kDa FITC-dextran and 40 kDa TRITC-dextran transfer are shown for high and low doses of IFN-γ compared to untreated controls. All error bars represent standard error of the mean. A one-way ANOVA with α = 0.05 was utilized to compare dose responses within a group unique to each donor, cytokine dose curve, and tracer. Post hoc analyses were performed with Tukey’s multiple comparisons test after ANOVA. (ns not significant, * *p* < 0.05, ** *p* < 0.01, *** *p* < 0.005, **** *p* < 0.0001. N = 4). (**c**) Following damage to colon micro-tissues, media was collected from microfluidic chambers and IL-8 was measured using a commercial ELISA assay kit. All error bars represent standard error of the mean. A one-way ANOVA was performed with an α = 0.05 and Dunnett’s multiple comparisons test was used to compare cytokine stimulation conditions to the untreated control for each donor. (ns not significant, * *p* < 0.05, ** *p* < 0.01, *** *p* < 0.005, **** p < 0.0001. N = 3).

All donor-derived tissues stimulated with IFN-γ at any dose demonstrated reduced barrier function as measured by increased transfer of a fluorescent tracer across the epithelial barrier (Fig. 5A). For each donor, both the low concentration and high concentrations of IFN-γ resulted in the same resulting percent transfer of tracer, suggesting that 0.1 U/mL was sufficient to maximize barrier permeability (Fig. 5B). The same result of maximal transfer at 0.1 U/mL was observed across all three sizes of tracer molecule. Similarly, an attempt to demonstrate synergy between IFN-γ and TNF-α by treatment with 0.1 U/mL IFN-γ plus 1 ng/mL TNF-α also resulted in similar tracer transfer (Supplementary Fig. S4). Overall, molecule permeability tests aligned with and complemented previous TEER measurements to afford consistent, facile assessment of donor-dependent colon micro-tissue barrier effects from damage stimuli. Notably, thorough evaluation of the mechanism of increased tissue permeability was not the goal of this work, however cytotoxicity levels were measured by lactate dehydrogenase (LDH) release (Supplementary Fig. S5). Overall, cell damage was quite low in these experiments, in the range of 0.5–2.0% of maximum, although significantly higher than background in some IFN-γ containing conditions. Future studies with these and other disrupter molecules could evaluate colon injury and recovery mechanisms using live/dead staining, other vital dyes such as Calcein, and evaluation of cell junction proteins, all of which are straightforward to evaluate in PREDICT96-cultured colon tissue^27^.

### Inflammatory cytokine stimulation effects on epithelial cell IL-8 secretion

IL-8, a neutrophil and T cell recruiting cytokine^49^, has been shown to be differentially regulated by cytokines TNF-α and IFN-γ in primary intestinal cells. Secreted protein analysis is easily performed in PREDICT96 colon micro-tissues, and sensitivity is further aided by the inherent low media volume-to-cell parameters of the platform, serving to concentrate the secreted factors and boost signal. As a straightforward example, acute IL-8 secretion post-cytokine insult was measured for all donor derived micro-tissues using ELISA performed on cell culture supernatant following a 12-h cytokine stimulation. Donor to donor variation in IL-8 secretion was identified in the response of colon micro-tissues to TNF-α stimulation (Fig. 4C). TNF-α exposure had no significant impact on IL-8 secretion of micro-cultures derived from Donor A; however, in cultures derived from Donor B and C, exposure to 1 ng/mL and 250 ng/mL of TNF-α increased IL-8 secretion strongly (Donor B) or moderately (Donor C) (B: *p* = 0.0001, *p* = 0.0003; C: *p* = 0.0378, *p* = 0.0371). IL-8 secretion for all donor derived colon micro-tissues was significantly impacted in response to IFN-γ stimulation (Fig. 5C). In Donors A and B, exposure to 0.1 U/mL and 50 U/mL IFN-γ significantly decreased IL-8 secretion, a result that parallels previous reports with primary human cells^43^, while Donor C tissue sensitivity was less significant. The highly reproducible IL-8 secretion results provide a consistent measure of epithelial sensitivity in response to IFN-γ and TNF-α.

## Discussion

We have developed a high-throughput primary colon-on-chip platform that enables screening of large numbers of compounds or dose curves in a relevant phenotypic assay. The selective permeability of the epithelial barrier is a key feature of the healthy colon, and increased permeability by larger molecules and pathogens is the cause and result of inflammatory diseases of the intestine^18,19,50^. Published intestine-on-chip platforms initially relied on immortalized cell lines, which typically have high TEER values compared to known in vivo biology, indicating potential limitation of their predictive capability, and primary cell integration is expected to improve the relevance of these model systems^51–53^. Primary cells derived from human proximal small intestine, including duodenum and jejunum, were the first intestinal microfluidic devices to be developed, and demonstrated the presence of relevant cell types and formation of the epithelial barrier^54,55^. More recently, integration of colorectal primary cells into devices has supported investigation of dietary and inflammatory molecule effects on human colon tissue^56–58^. In particular, these efforts have resulted in novel tissue culture devices that have allowed for identification of structural, stretch and flow cues that increase the relevant differentiation of colon cells within the device. However, these devices are highly specialized and typically support only single or small replicate numbers, rendering them less useful for studies that benefit from high throughput capacity, such as drug discovery. An example of a multiplexed system is the 40 chip device described by Beaurivage et al. directly comparing to Caco-2 cultures and demonstrating the benefits of utilizing primary colon spheroids as a relevant cell source^56^. Functional analysis of epithelial barrier function and secretion analysis across multiple colon donors has not been pursued in high throughput MPSs. The PREDICT96 colon model combines primary human colon cells, and a high throughput framework with integrated pumping and TEER measurements that enable the first system to efficiently and sensitively assess permeability across multiple donors and many dose treatments. This system could be used to identify barrier protective therapeutics, by inducing TNF-α and IFN-γ mediated barrier damage and screening for factors that decrease the permeability damage.

Prior to pursuing cytokine studies, colon micro-tissues were characterized to ensure the growth of physiologically representative monolayers from diverse donor sources in PREDICT96 microfluidic devices. Tissue phenotype was characterized utilizing qRT-PCR, immunofluorescence imaging, and barrier function as measured by TEER. Changes in gene and protein expression indicated that relevant, differentiated tissue was formed during the 7-day culture of colon cells within the PREDICT96 devices, and in particular, markers of small intestine-specific cells, APOA, LYZ, REG3A, and SI were decreased along with markers of stem-like cells (ASCL2, KI67, LGR5, OLFM4). Genes coding for complex mucus proteins such as gel forming mucus (MUC2, MUC5AC) and transmembrane mucin proteins (MUC1, MUC4) were significantly upregulated in colon micro-tissues compared to ileum sourced micro-tissues. Tight junction organization through ZO-1 stain localization to cell borders demonstrated cobblestone morphology in colon micro-tissues and this correlated with a robust increase in TEER after change to this media at Day 4. TEER values of colon micro-tissues ranged between 300 and 500 Ω cm^2^, lower than Caco-2 monolayers, which typically report TEER above 1000 Ω cm^2^, although it is not possible to compare these values to in vivo tissues, given the difficulty of separating epithelium from underlying lamina propria^36,42,59^. Taken together, these results indicate that the colon-derived progenitor cells retained their ability to differentiate into colon-like micro-tissues.

To characterize the PREDICT96 colon-on-chip ability to emulate a diseased intestinal epithelium, TNF-α and IFN-γ were selected as damage-inducing cytokines due to their relevance to IBD etiology. High levels of both cytokines are associated with the disease state, and anti-TNF therapeutics are effective at treating IBD^60–62^. In clinical studies, patient sera samples collected in active cases of both Crohn’s disease (CD) and ulcerative colitis (UC) contained upregulated levels of TNF-α and IFN-γ with 380-fold and 640-fold increases respectively when compared to healthy donor sera^63^. In addition to having clinical relevance, TNF-α and IFN-γ have been heavily characterized through in vitro research as damage agents that impact both barrier function and IL-8 secretion from intestinal epithelial cells^37–41^. In the studies presented here, the high throughput nature of PREDICT96 allowed combinatorial testing of TNF-α and IFN-γ in combinatorial dose curves and revealed potentially synergistic effects of these two factors on colon micro-tissue TEER. These results highlight the extent of data that can be collected in a single experiment with PREDICT96, while employing a relevant primary cell model and a complex phenotypic readout.

TEER evaluates the permeability of ions through paracellular and transcellular paths. To evaluate permeability to larger moieties, a molecular permeability assay was established. Using high and low concentrations of TNF-α and IFN-γ based on the TEER study, the barrier function of colon-microtissues from three donors were further studied with a molecular permeability assay. In this case, fewer cytokine conditions allowed for 3 replicates of each condition within a single experiment. The sensitivity and precision of the permeability assay were highlighted by reproducible responses of donor-specific micro-tissues when treated with inflammatory cytokines. Micro-tissues derived from Donor A were insensitive to TNF-α at 1 and 250 ng/mL as measured by permeability and IL-8 secretion. This verified Donor A’s previous sustained TEER in conditions with high doses of TNF-α (Fig. 3). Micro-tissues derived from Donors B and C showed nuanced sensitivity to TNF-α, where those derived from Donor B appear to be more sensitive to TNF-α damage than micro-tissues derived from Donor C in this experiment. Lucifer Yellow dye (0.4 kDa) and 4 kDa dextran can transfer across a membrane when leak paths between cells form as intercellular connections break down, and also when cells die creating an unrestricted pathway across the barrier. Transport of the 40 kDa dextran is more constrained by size and occurs through unrestricted pathways^18,64^. The assays developed here show sensitivity across this range of molecule sizes, where the larger molecules consistently measured as lower transfer across the barrier. This sensitivity can be used for nuanced readout of barrier damage between donors and doses. Micro-tissues treated with IFN-γ across all donors resulted in consistent permeability readouts same at both 0.1 and 50 U/mL IFN-γ, suggesting that 0.1 U/mL is sufficient to achieve the maximal damage possible within the experimental parameters. This is in contrast to the TEER readout, where sub-maximal effect was measurable into the range of 10 s of U/mL and emphasizes that electrical resistance and molecular permeability measure distinct aspects of the barrier function (Figs. 3, 5). In parallel with their relative size, Lucifer Yellow had the highest maximum transport at about 30%, 4 kDa dextran next at about 20% and 40 kDa dextran the lowest transfer of about 12–15% to the bottom channels after 6 h. These maxima provided a sufficient window to identify significant variation in permeability between TNF-α doses (Fig. 4B). The reproducibility of transport level within a condition is striking, and it is possible that this is driven by the recirculation in both top and bottom chambers throughout the assay time frame, allowing for consistent concentration of dye at apical and basal surfaces. Interestingly, low LDH levels indicate that cell death was minimal, and future studies could evaluate the mechanism by which barrier permeability is increased to an extent that permits larger molecular transfer. These results highlight that this assay provides a powerful tool to analyze reproducibly and precisely colon permeability and sensitivity to damage.

Significant downregulation of IL-8 was measured in response to IFN-γ stimulation, a response that has previously been shown in primary intestinal epithelial cells^40,60^ (Fig. 5C). In contrast, IFN-γ has been previously shown to significantly upregulate IL-8 secretion in HT29 cells, directly contradicting the response of primary IECs^39^. These findings collectively support the biological importance of utilizing primary intestinal cells within research avenues surrounding intestinal disease states. The PREDICT96 device allowed direct comparison of IL-8 production responses across micro-cultures derived from 3 donors. Findings confirmed the inhibitory effect of IFN-γ, but not TNF-α, on IL-8 secretion from epithelial monolayers. Combined doses of TNF-α and IFN-γ resulted in significant decreases in IL-8 secretion in Donor A and Donor B, while Donor C micro-tissue IL-8 secretion was not significantly impacted (Supplementary Fig. S4).

By combining primary intestinal colon cultures with PREDICT96, a 96-device arrayed bilayer microfluidic platform, we have demonstrated the capability to study physiologically relevant human intestinal biology in a modality that can support the bandwidth required for applications such as therapeutic and small molecule screening. Utilizing multiplexed TEER sensing and non-destructive tracer permeability readouts, barrier function was studied across multiple tissue donors in response to TNF-α and IFN-γ stimulations. Differences in donor epithelial barrier response among primary colon micro-tissues was observed and supplemented by a preliminary evaluation of epithelial cytokine response through IL-8 secretion characterization. The ability to test unique epithelial barrier-based donor responses to stimulatory and therapeutic agents in such high throughput is a powerful and enabling capability for the field of IBD research and other intestinal diseases. These colon micro-tissues that mimic in vivo biology would benefit from continued evaluation expanding the complexity of the model. Increased donor diversity and the incorporation of innate immune components known to be associated with colon-specific inflammation and disease states could expand the scientific questions to be explored within the context of the model presented in this study. Moreover, expanding this robust damage model to other known epithelial chemokines would further demonstrate physiological relevance of these PREDICT96 enabled colon micro-tissues.

## Methods

### Ethics approval and informed consent

All experiments in this manuscript were carried out at Draper, and all experimental protocols and the use of human-derived cells were reviewed and approved by Draper’s Institutional Biosafety Committee (institutional and/or licensing committee). All methods were carried out in accordance with the guidelines and regulations approved by Draper’s Institutional Biosafety Committee. Experiments in this manuscript use cells isolated from colon segments from de-identified deceased human donors. Colon segments were obtained from the International Institute for the Advancement of Medicine (IIAM). IIAM only receives non-transplantable organs/tissues from one of the 56 Organ Procurement Organizations (OPO) in the U.S. that are authorized for medical research and education. OPOs operate under a set of standards established by the Association of Organ Procurement Organizations (AOPO) and UNOS. These OPOs refer non-transplantable organs and tissues for placement with medical research where proper authorization for medical research and education has been obtained and which is documented on each OPO’s Authorization Form. Each OPO operates individually under its own protocol to obtain authorization. These SOPs must conform to each respective state’s Uniform Anatomical Gift Act (UAGA) as well as the hospitals’ policies in each region. All of the specimens distributed through IIAM are obtained from such OPOs, all of whom are non-profit organizations who are legally mandated to obtain authorization from the legal next-of-kin in accordance with each state’s UAGA, or abide by First Person Consent as documented in a Donor Registry. The authorized gifts are distributed, through IIAM, to medical research entities that have completed IIAM’s Biomaterial Transfer Agreement. An Application Review Committee approves of all requested biomaterials based on the MTF/IIAM Tissue Use Policy.

### Primary intestinal epithelial spheroid isolation and culture

Colon segments from de-identified deceased human donors were obtained from the International Institute for the Advancement of Medicine (IIAM). Donors did not have a history of IBD or other known intestinal disease. While the donors include diversity of age, gender and race, this study is not meant to address potential correlations with genetics or background (Supplementary Table 1). Crypt-isolated spheroid culture was established as previously described^24,65,66^. In brief, short (~ 4–6 inch) segments of human colon were rinsed and dissected to small pieces, and a small subset used for further isolation. Selected tissue was further broken down with scalpels and washes in DTT and EDTA. Upon release of crypts from the tissue, they were collected, washed and embedded in Matrigel for long term culture. Established colon spheroid cultures were passaged weekly and maintained in undiluted Matrigel (Corning Inc., Corning, NY) domes supplemented with proliferation media (PM; formulated as previously described^27^) every other day following established protocols^24,65^. In brief, weekly passaging consisted of collecting Matrigel domes and breakdown of spheroids with trypsin. After washing, cell pellets were resuspended in Matrigel and plated into 24 well plates as 15uL/well domes; solidified domes were submerged in PM, which was changed every 2 days. All cultures were maintained in humidity-controlled incubators at 37 °C with 5% CO_2_.

### Colon micro-tissue seeding and maintenance in PREDICT96

Primary colon cells and PREDICT96 plates were prepared as previously described, and colon cell suspension was introduced into the top microfluidic chamber^27^. In brief, PREDICT96 plates were O_2_ plasma treated for 5 min, and then channels were washed with 70% ethanol. After aspirating the ethanol and rinsing channels with phosphate buffered saline (PBS), Matrigel (Corning Inc., Corning, NY) was diluted 1:160 in ice cold PBS and delivered to the top microfluidic channel of each device. Matrigel coated channels polymerized at 37 °C for at least 1 h prior to seeding. After polymerization, media was added to the top channel of the device to dilute the 1:160 Matrigel solution. To achieve a cell suspension, colon spheroids were processed by enzymatic and mechanical dissociation methods as previously described and were seeded into PREDICT96 devices at a cell density of approximately 1.6 × 10^6^ cells/cm^2^, resulting in approximately 60,000 cells per microfluidic device^27^. Additional media was added to the top chambers of devices after cell delivery to the membrane. Seeded plates were maintained in a humidified cell culture incubator at 37 °C with controlled 5% CO_2_ for the duration of culture. PM media was replenished every other day during initial stages of culture. On Day 4, media was replenished with differentiation media (DM; formulated as previously described^27^) for the duration of the culture timeline while undifferentiated controls were maintained in PM. Previously reported calcein staining indicated that cells remain viable at 7 days following seeding into devices^27^.

### Ileum micro-tissue seeding and maintenance in PREDICT96

Primary ileum epithelial stem cells were isolated and cultured using methods identical to those described for colon cells. Seeding into PREDICT96 was also performed using the same methods described for colon, including culture timelines. Detailed results other than those reported here will be described elsewhere.

### Transepithelial electrical resistance measurements

We quantified micro-tissue barrier integrity by measuring longitudinal TEER readings every day up to and including day 14 of culture (cells were seeded on day 0). We used our custom MPS TEER system connected to an Epithelial Volt/Ohm Meter (EVOM2, from World Precision Instruments, Sarasota, FL) as previously described^27^. Plates were removed from the incubator and equilibrated to room temperature for 15 min prior to resistance measurement to ensure stable readings, since stable TEER is dependent on temperature. TEER measurements were collected outside of the incubator in an aseptic biosafety cabinet. On days media was replenished, TEER measurements were taken prior to media replenishment to minimize any measurement artifacts.

### Treatment of micro-tissues treatment with pro-inflammatory cytokines

Colon monolayers were cultured in devices with 4 days of growth in PM, followed by 3 days of growth in DM. Mature, differentiated colon micro-tissues were exposed to pro-inflammatory cytokines TNF-α and IFN-γ (Peprotech) at various concentrations as indicated in the figures. Cytokine solutions were introduced on day 7 of culture prior to endpoint assays and collections performed on day 8 unless otherwise specified. Stock solutions of cytokines were prepared as recommended by the supplier and were diluted in DM prior to treatment. Colon micro-tissues were incubated with cytokine treatments in both top and bottom chamber for 24 h for initial TEER studies and 12 h for barrier permeability studies prior to supernatant collection and fluorescent probe-based molecular permeability measurements.

### Supernatant sampling and analysis

To assess IL-8 chemokine secretion by colon micro-tissues in response to varying doses of TNF-α and IFN-γ treatments, supernatants were collected from the top and bottom chambers of devices after 12 h of incubation with the previously described cytokine conditions. Media collected from top and bottom chambers were pooled and then sampled for analysis. The IL-8 ELISA was performed on micro-tissue supernatant samples diluted 1:10 and following supplier’s instructions (R&D Systems, Minneapolis, MN).

The same collected media samples were used to evaluate cytotoxicity through Lactate Dehydrogenase activity. Using the CyQUANT LDH Cytotoxicity Assay Kit (Invitrogen/Thermo Fisher), supernatants were evaluated following the protocol provided. Maximum LDH Activity was measured from 4 individual wells, following the protocol methods. Resulting measurements were background subtracted, averaged and normalized to the average maximum cytotoxicity readout, yielding the reported % maximum cytotoxicity that is reported.

### Macromolecular permeability assay

Biomolecule permeability was measured across colon micro-tissues with a fluorescent tracer-based permeability assay. Prior to the start of the permeability assay, colon micro-tissues were stimulated for 12 h with the addition of pro-inflammatory cytokines, TNF-α and IFN-γ, which have previously been shown to impact barrier function in vitro^28,67^. A triplex test compound solution was used to assess permeability by diversely sized molecules conjugated to different fluorescent dye tracers within the same device. This triplex solution consisted of Lucifer Yellow salt with a molecular weight of 0.4 kDa (Sigma, Millipore Sigma), a Fluorescein isothiocyanate (FITC)-tagged dextran with a molecular weight of 4 kDa (Sigma, Millipore-Sigma), and a Tetramethylrhodamine (TRITC)-tagged dextran with a molecular weight of 40 kDa (Sigma, Millipore-Sigma). The three tracers were received and reconstituted following supplier’s instructions as separate dyes at stock concentrations of 20 mM, 6.25 mM and 6.25 mM for Lucifer Yellow (LY), 4 kDa FITC-dextran, and 40 kDa TRITC-dextran, respectively. The stock solutions were then combined and diluted to a working concentration of 100 μM for each tracer (LY: 1:200, 4 kDa FITC-dextran: 1:62.5, and 40 kDa TRITC-dextran: 1:62.5) in Hank’s Balanced Salt Solution (HBSS) before being added to the top chambers of devices. The final solution in the top chamber was an HBSS tracer solution containing 100 μM LY, 100 μM 4 kDa FITC-dextran, and 100 μM 40 kDa TRITC-dextran. An equivalent volume of blank HBSS buffer was added to the bottom chamber, and both top and bottom chamber solutions were recirculated using the onboard pumping system at a flow rate of 60 uL/hour to maintain well mixed tracers throughout the assay. After 6 h, fluidic samples were taken from the top and bottom chambers to be quantified on a fluorescent plate reader (Synergy H1 Microplate Reader) following an 8-point standard curve at excitation and emission values of 420/530 nm, 490/530 nm, and 540/577 nm for Lucifer Yellow (LY), 4 kDa FITC-dextran, and 40 kDa TRITC-dextran, respectively.

Tracer concentrations in the top and bottom chambers after 6 h were related by the following equation:

$$\% bottom = \frac{{\left[ {Tracer_{bottom} } \right]}}{{\left[ {Tracer_{bottom} } \right] + \left[ {Tracer_{top} } \right]}}*100$$

### Immunofluorescence microscopy

The PREDICT96 platform affords high-fidelity staining and imaging in situ with key antibodies to characterize specialized cell types and features within the differentiated colon micro-tissues. Differentiated micro-tissues were established over 7 days, and then fixed at room temperature with 4% paraformaldehyde solution (Thermo Fisher) for 15 min. After fixation, cells were permeabilized with 0.1% Triton-X 100 (Millipore-Sigma) for 15 min and blocked with 3% bovine serum albumin (BSA) for an hour at room temperature (Millipore-Sigma). Primary antibodies were added to devices and incubated overnight at 4 °C (MUC-2 (ThermoFisher #MA5-12345), ZO-1 (ThermoFisher #339100), Ezrin (BD Biosciences #610602)). Tissues were then incubated with secondary antibodies (Goat α-Mouse Alexa Fluor™ 488 (ThermoFisher #A-11001), Alexa Fluor™ 647 Phalloidin (ThermoFisher #A22287), Hoechst 33342 (ThermoFisher #62249) for 3 h at room temperature protected from light. All incubations were performed on a rocker, and wash steps were performed three times with PBS between each of the aforementioned incubations. Devices were visualized and imaged *in-situ* on an LSM 700 Zeiss AxioObserver confocal microscope, using a Zeiss LD C-apochromat 40x/1.1 W Korr M27 lens. Settings are shown below; in cases where variation in settings occurred between samples, each setting is listed, and the related sample is indicated by the unique stain for that sample (MUC2, EZRIN or ZO-1) and all samples also included Phalloidin and Hoechst stains. Zen Black software (Zeiss; Version 2012 SP5) was used to add 20 µm scale bars.

**Table:** 

Scaling (per pixel)	MUC2	0.10 µm × 0.10 µm × 1.01 µm		
	EZRIN	0.10 µm × 0.10 µm × 0.86 µm		
	ZO-1	0.10 µm × 0.10 µm × 1.14 µm		
Image size (pixels)		1024 × 1024		
Image size (scaled)		106.70 µm × 106.70 µm		
Bit depth		16 Bit		
		Track 1	Track 2	Track 3
Pinhole	MUC2	1.09 AU	1.14 AU	1.11 AU
	EZRIN	1.09 AU	1.14 AU	1.11 AU
	ZO-1	0.85 AU	1.14 AU	1.84 AU
Laser wavelength	MUC2	639 nm: 17.43%	488 nm: 15.43%	405 nm: 79.97%
	EZRIN	639 nm: 17.43%	488 nm: 24.13%	405 nm: 79.97%
	ZO-1	*Not captured*		
Scan zoom		1.5		
Pixel time		3.15 µs		
Line time		30.00 µs		
Channel color		Magenta	Green	Blue
Excitation wavelength		639	488	405
Emission wavelength		660	518	450
Detection wavelength		644–800	300–550	420–480
Binning mode		1 × 1		
Detector gain	MUC2	694.9	621.0	676.5
	EZRIN	648.1	679.4	676.5
	ZO-1	700.0	700.0	750.0
Detector digital gain		1.0		

### Quantified gene expression

To characterize the gene expression of micro-tissues established in devices, messenger ribonucleic acid (RNA) was collected from various growth and treatment conditions for analysis via quantitative reverse transcription polymerase chain reaction (qRT-PCR). RNA samples were collected prepared each from a single PREDICT96 device after tissue lysis via RLT buffer (Qiagen Mini Prep Kit) with 1% β-mercaptoethanol to inhibit nuclease activity. Samples from microfluidic chambers underwent a 5 min incubation of with RLT which was then collected. One additional RLT wash was performed to collect all residual RNA. A single device yielded enough mRNA for downstream analysis thus devices were not pooled. The mRNA was isolated and purified according to manufacturer’s guidelines. Prior to moving forward with cDNA synthesis, RNA quality and quantity was measured on a 4200 Tapestation system. All RNA samples utilized had RINe values greater than 9.5. cDNA synthesis and qPCR amplification was performed utilizing TaqMan kits and probe sets (ThermoFisher, Table 2), following manufacturer-recommended protocols. Ct curves and values were used to compare treatment conditions with comparative Ct analysis. All sample datasets represent relative expression of fold induction to housekeeper gene β-actin.

**Table 2:** TaqMan assays used.

Target gene	Gene ID	Assay catalog number	Cell type/process
Achaete-scute family bHLH transcription factor 2	ASCL2	Hs00270888_s1	Stem cell
Leucine rich repeat containing G protein-coupled recptor5	LGR5	Hs00969422_m1	Stem cell
Marker of proliferation Ki-67	MKI67	Hs01032434_ml	Proliferation
Olfactomedin 4	OLFM4	Hs00197437_ml	Stem cell
Apolipoprotein A4	APOA4	Hs00166636_ml	Ileum enterocyte
Lysozyme	LYZ	Hs00426232_ml	Antimicrobial
Regenerating family member 3 alpha	REG3A	Hs00170171_ml	Paneth cell
Sucrase isomaltase	SI	Hs00356112_ml	Ileum enterocyte
Mucin 1, cell surface associated	MUC1	Hs00159357_ml	Membrane-bound mucin
Mucin 2, oligomeric mucus/gel-forming	MUC2	Hs03005103_g1	Goblet cell
Mucin 4, cell surface associated	MUC4	Hs00366414_ml	Goblet cell
Mucin 5AC, oligomeric mucus/gel-forming	MUC5AC	Hs01365616_ml	Goblet cell

### Statistical analysis

All statistical analyses were performed using GraphPad Prism 9.3.1. Graphs represent sample means and error bars reflect standard error of the mean. Statistical significance is represented by asterisks on relevant plots. For RT-PCR analysis, either a two-tailed unpaired t-test or Mann–Whitney test was used to analyze tissues grown in PM versus DM with an α = 0.05. For permeability analyses, a one-way ANOVA with α = 0.05 was utilized to compare dose responses within a group unique to each donor, cytokine dose curve, and tracer. Post hoc analyses were performed with Tukey’s multiple comparisons test after ANOVA. For data display, all three tracers were displayed included on bar charts together though statistical comparisons between tracer sizes were not performed. For ELISA analysis, a one-way ANOVA was performed with an α = 0.05 and Dunnett’s multiple comparisons test was used to compare cytokine stimulation conditions to the untreated control for each donor.

## Supplementary Information

Supplementary Information.

## Data Availability

Any datasets generated during the current study and not presented herein are available from the corresponding author on reasonable request.
