# Supplementary material for: A platform to reproducibly evaluate human colon permeability and damage
Source: Sci Rep. 2023 Jun 1;13:8922. doi: 10.1038/s41598-023-36020-8 (PMC10235044; doi:10.1038/s41598-023-36020-8)
Supplement: Supplementary file 1 — Supplementary Information. [file 41598_2023_36020_MOESM1_ESM.pdf]

A platform to reproducibly evaluate human colon permeability and damage

Elizabeth E. Marr[1], Thomas J Mulhern[1], Michaela Welch[1], Philip Keegan[1], Celia Caballero-Franco[2], Bryce G. Johnson[2], Marion Kasaian[2], Hesham Azizgolshani[1], Timothy Petrie[1], Joseph Charest[1], Elizabeth Wiellette[1]*

1 Draper, 555 Technology Sq., Cambridge, MA 02139, USA

2 Pfizer Inflammation and Immunology, 1 Portland St., Cambridge, MA, 02139, USA

*Corresponding author: ewiellette@draper.com

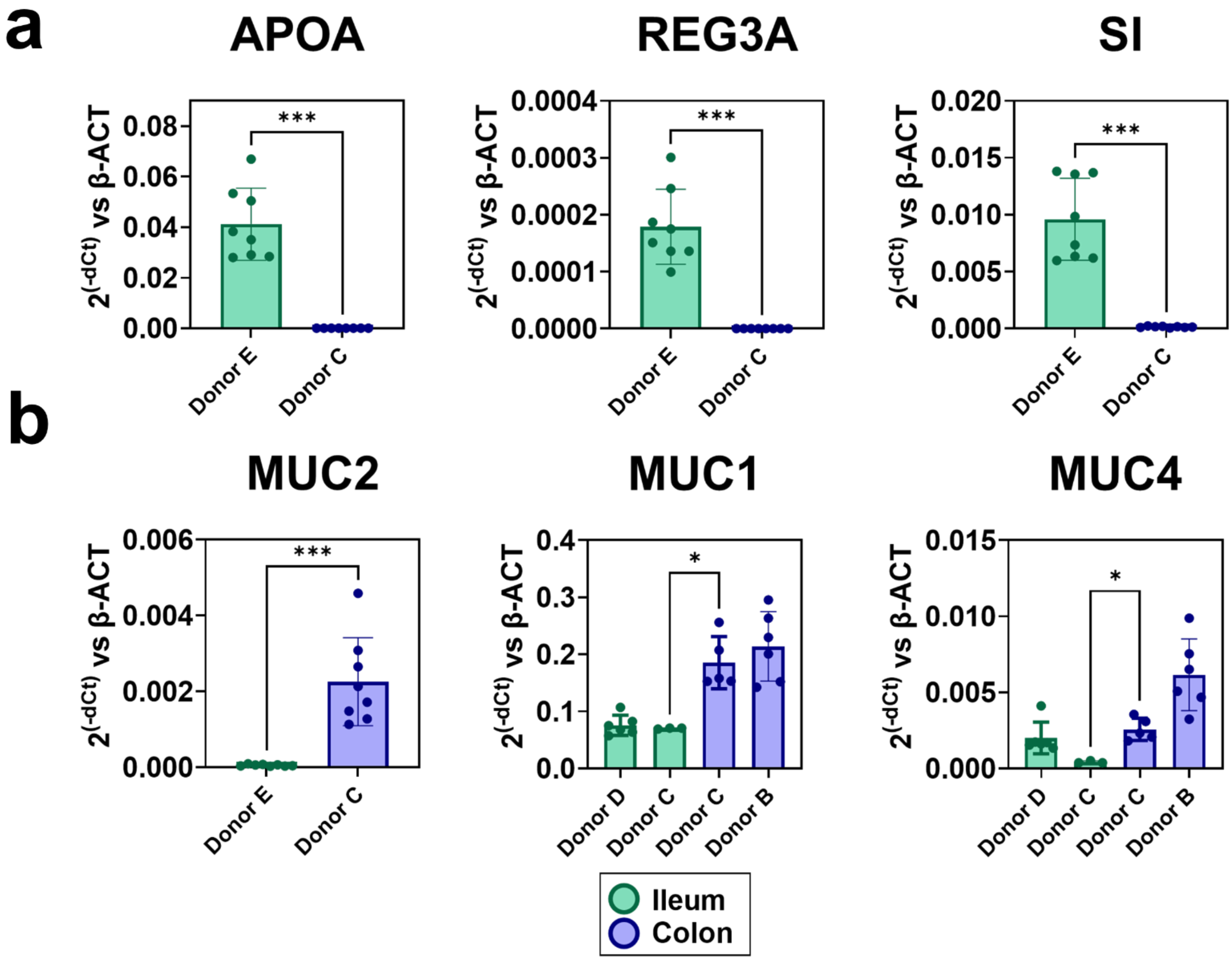

S1. Differential gene expression in ileum and colon microtissues in PREDICT96. Differentiation markers between colon and ileum micro-tissues was compared by changes in gene expression of key tissue specific markers (ileum: APOA, LYZ, REG3A, SI; colon: MUC2, MUC5AC, MUC1, MUC4). Quantitative RT-PCR measurement of gene expression is reported as relative to expression of the housekeeping gene β-ACTIN for each sample. In all cases, error bars represent standard error of the mean. A two-tailed unpaired t-test was used to analyze tissues grown in PM versus DM with an α = 0.05 (* $p<0.05$, ** $p < 0.01$, *** $p < 0.005$, **** $p< 0.0001$). N = 3-8.

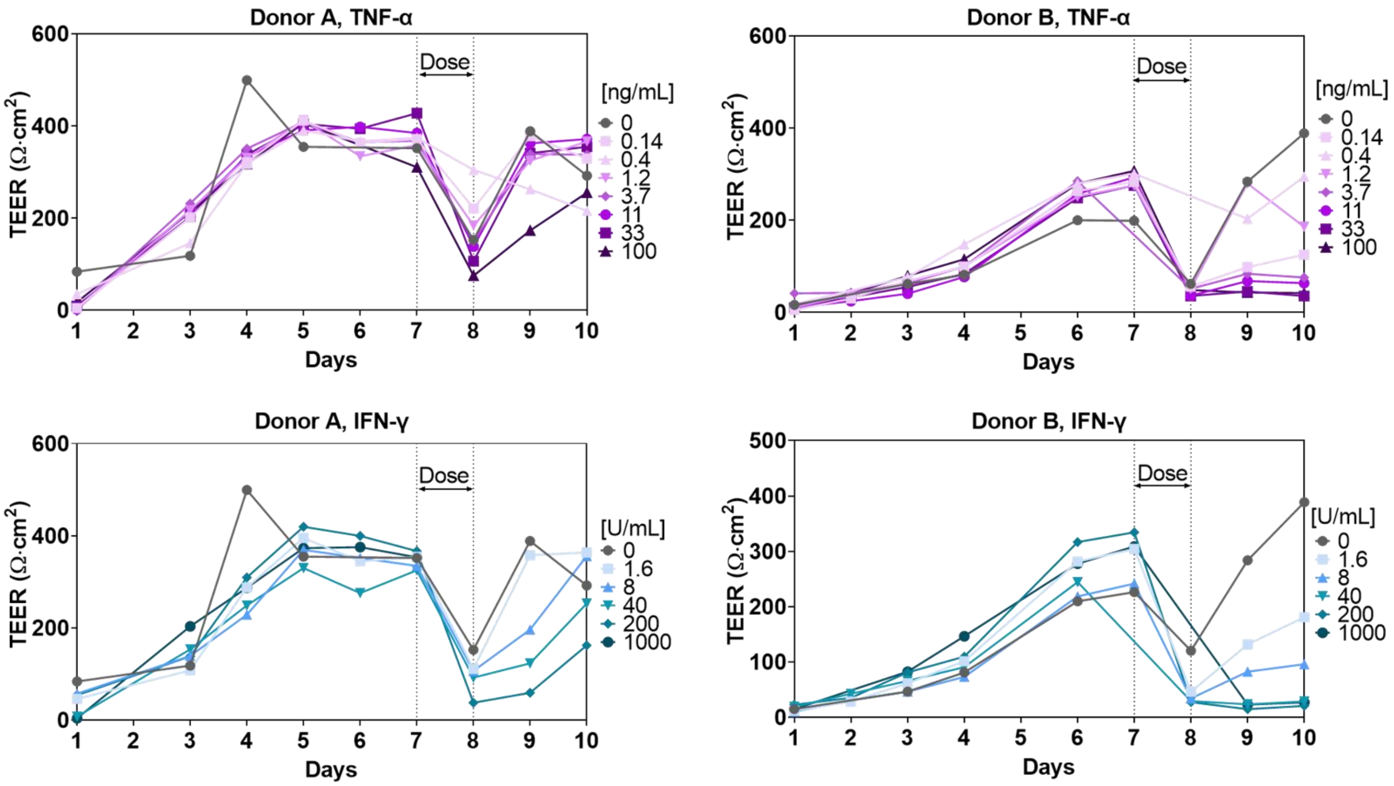

Figure S2: Response of colon epithelial monolayers to cytokine exposure. Epithelial monolayers developed TEER over 7 days, and then were treated with cytokines for 24 hours, washed, and allowed to recover for an additional 48 hours. TEER was measured daily over the course of the experiment, indicated by development of a monolayer with consistent resistance by day 5/6. Note that day 5 data were not collected for Donor B in this case. During the period of cytokine treatment and closely spaced TEER measurements, the TEER dropped (day 7-8), but then recovered following removal of cytokines.

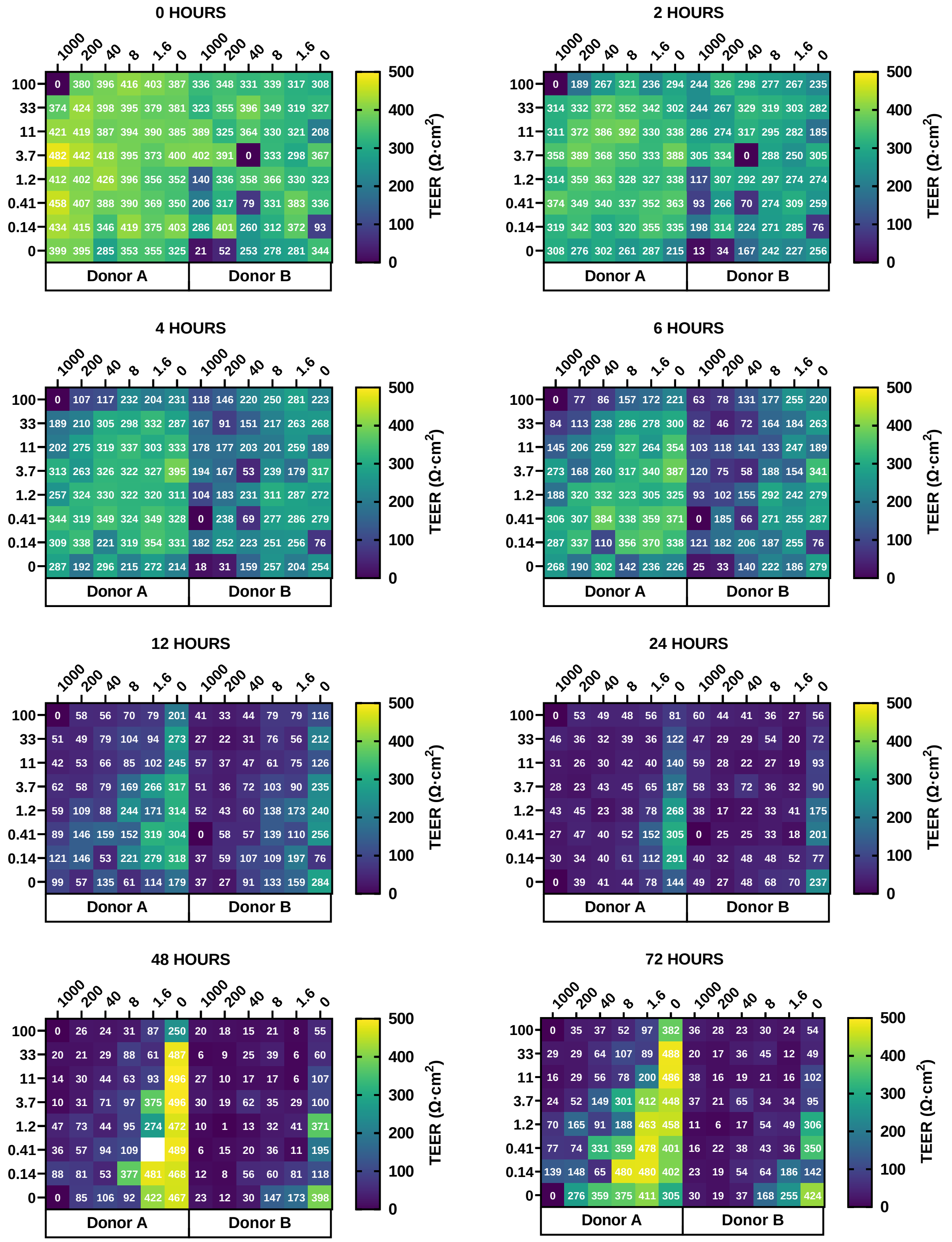

Figure S3: Heat maps of TEER measured across the entire period of damage and recovery. TEER measurements were collected throughout the period of damage (0-24 hr) and recovery (up to 72 hr) as outlined in Figure 3. Time points for each heat map are indicated above the grid. Relative TEER is highlighted as indicated by the range map on the right of each grid, and exact TEER for each well is indicated in white numbers within each well.

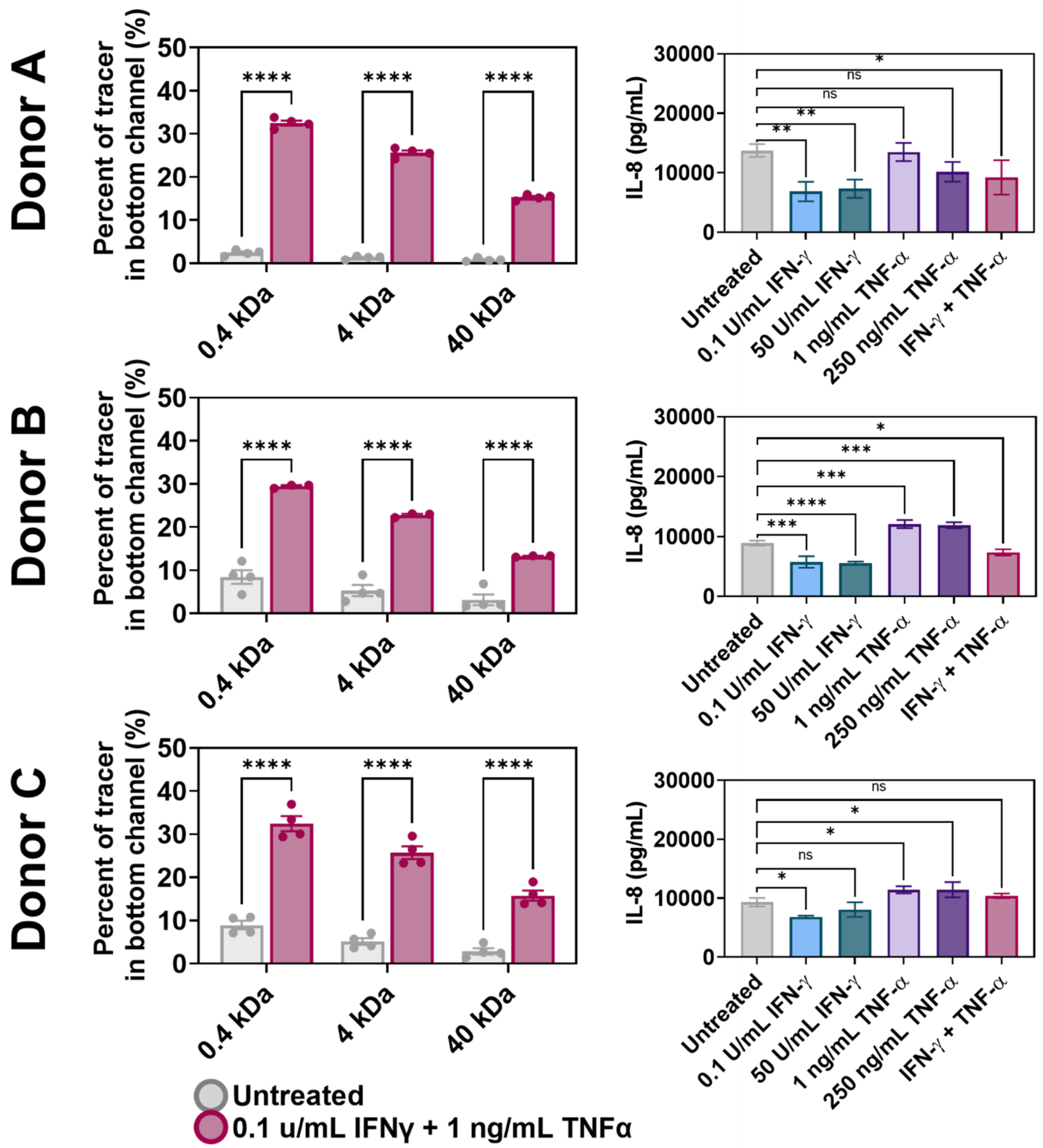

Figure S4. Combined TNF-α and IFN-γ dosing impacts colon micro-tissue permeability and IL-8 secretion a) Established colon micro-tissues (verified by high TEER) were exposed for 12 hours to IFN-γ doses and then replaced with the tracer dye molecules under recirculation. Permeability was assayed as fluorescent tracer transfer from top to bottom channel after 6 hours. Values are displayed as percent of tracer in bottom channel to quantify probe transfer across the epithelial barrier of micro-tissues derived from 3 colon donors. Results of 0.4 kDa lucifer yellow (LY), 4 kDa FITC-dextran and 40 kDa TRITC-dextran transfer are shown for high and low doses of IFN-γ compared to untreated controls. All error bars represent standard error of the mean. A one-way ANOVA with $\alpha = 0.05$ was

utilized to compare dose responses within a group unique to each donor, cytokine dose curve, and tracer. Post hoc analyses were performed with Tukey's multiple comparisons test after ANOVA. (ns not significant, * $p<0.05$, ** $p < 0.01$, *** $p < 0.005$, **** $p< 0.0001$. N = 4). (b) Following damage to colon micro-tissues, media was collected from microfluidic chambers and IL-8 was measured using a commercial ELISA assay kit. All error bars represent standard error of the mean. A one-way ANOVA was performed with an $\alpha = 0.05$ and Dunnett's multiple comparisons test was used to compare cytokine stimulation conditions to the untreated control for each donor. (ns not significant, * $p<0.05$, ** $p < 0.01$, *** $p < 0.005$, **** $p< 0.0001$. N = 3).

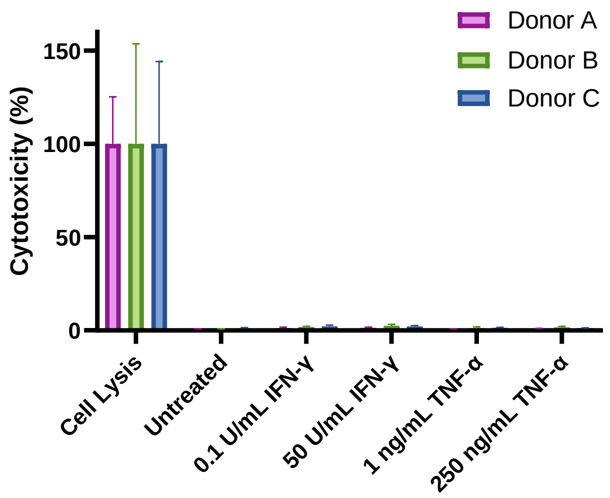

	Donor A			Donor B			Donor C		
	Mean	SD	p-value	Mean	SD	p-value	Mean	SD	p-value
Cell Lysis	100.00	25.26	2.21e-004	100.00	53.70	0.01	100.00	44.14	4.20e-003
Untreated	0.43	0.50		0.50	0.36		1.15	0.44	
0.1 U/mL IFN-γ	1.26	0.49	0.06	1.81	0.45	3.92e-003	2.21	0.59	0.03
50 U/mL IFN-γ	1.40	0.45	0.03	2.52	0.78	3.25e-003	2.09	0.53	0.03
1 ng/mL TNF-α	0.72	0.14	0.32	1.25	0.63	0.08	1.40	0.24	0.37
250 ng/mL TNF-α	0.81	0.27	0.23	1.64	0.60	0.02	1.22	0.22	0.80

Figure S5: Lactate Dehydrogenase (LDH) assay as a measure of cell damage/death following TNFα and IFNγ treatment. Media collected from the permeability studies and analyzed for IL-8 secretion was also evaluated for cytotoxicity by LDH assay and is reported as a percentage of the maximum cytoxicity measured by lysing all cells in a device. Average (mean) % cytotoxicity is reported, including the standard deviation. The significance of cytotoxicity compared to the untreated condition was evaluated by two-way ANOVA and the P value is reported for each treated condition. A note that the cell lysis maximum cytotoxicity was not able to be performed on the Donor A cells, due to lack of sufficient cells for seeding. The maximum cytotoxicity for Donor A was generated by averaging the Donor B and C results.

Supplementary Table 1: Donor demographics. Age is given in years, and Body Mass Index (BMI) is provided.

**Donor**	**Gender**	**Age**	**BMI**
A	F	68	30
B	M	21	24
C	F	46	27
